# Subsidised housing and diabetes mortality: a retrospective cohort study of 10 million low-income adults in Brazil

**DOI:** 10.1136/bmjdrc-2022-003224

**Published:** 2023-06-22

**Authors:** Renzo Flores-Ortiz, Rosemeire L Fiaccone, Alastair Leyland, Christopher Millett, Thomas Hone, Maria Inês Schmidt, Andrêa J F Ferreira, Maria Y Ichihara, Camila Teixeira, Mauro N Sanchez, Julia Pescarini, Estela M L Aquino, Deborah C Malta, Gustavo Velasquez-Melendez, Juliane Fonseca de Oliveira, Peter Craig, Rita C Ribeiro-Silva, Mauricio L Barreto, Srinivasa Vittal Katikireddi

**Affiliations:** 1 Center for Data and Knowledge Integration for Health (CIDACS), Gonçalo Moniz Institute, Oswaldo Cruz Foundation (FIOCRUZ), Salvador, Bahia, Brazil; 2 Institute of Mathematics, Federal University of Bahia, Salvador, Brazil; 3 University of Glasgow, Glasgow, UK; 4 Public Health Policy Evaluation Unit, Department of Primary Care and Public Health, School of Public Health, Imperial College London, London, UK; 5 Faculty of Medicine, Federal University of Rio Grande do Sul, Porto Alegre, Brazil; 6 Tropical Medicine Center, University of Brasilia, Brasília, Brazil; 7 Institute of Collective Health, Federal University of Bahia, Salvador, Brazil; 8 Escola de Enfermagem, Universidade Federal de Minas Gerais, Belo Horizonte, Brazil; 9 Center of Mathematics of University of Porto (CMUP), University of Porto, Porto, Portugal; 10 MRC/CSO Social and Public Health Sciences Unit, University of Glasgow, Glasgow, UK; 11 School of Nutrition, Federal University of Bahia, Salvador, Brazil

**Keywords:** Epidemiology, Public Health, Observational Study, Mortality

## Abstract

**Introduction:**

Housing-related factors can be predictors of health, including of diabetes outcomes. We analysed the association between subsidised housing residency and diabetes mortality among a large cohort of low-income adults in Brazil.

**Research design and methods:**

A cohort of 9 961 271 low-income adults, observed from January 2010 to December 2015, was created from Brazilian administrative records of social programmes and death certificates. We analysed the association between subsidised housing residency and time to diabetes mortality using a Cox model with inverse probability of treatment weighting and regression adjustment. We assessed inequalities in this association by groups of municipality Human Development Index. Diabetes mortality included diabetes both as the underlying or a contributory cause of death.

**Results:**

At baseline, the mean age of the cohort was 40.3 years (SD 15.6 years), with a majority of women (58.4%). During 29 238 920 person-years of follow-up, there were 18 775 deaths with diabetes as the underlying or a contributory cause. 340 683 participants (3.4% of the cohort) received subsidised housing. Subsidised housing residents had a higher hazard of diabetes mortality compared with non-residents (HR 1.17; 95% CI 1.05 to 1.31). The magnitude of this association was more pronounced among participants living in municipalities with lower Human Development Index (HR 1.30; 95% CI 1.04 to 1.62).

**Conclusions:**

Subsidised housing residents had a greater risk of diabetes mortality, particularly those living in low socioeconomic status municipalities. This finding suggests the need to intensify diabetes prevention and control actions and prompt treatment of the diabetes complications among subsidised housing residents, particularly among those living in low socioeconomic status municipalities.

**WHAT IS ALREADY KNOWN ON THIS TOPIC**

- Access to housing is a current global social challenge and also a predictor of health. Subsidised housing has been a key social policy to support low-income populations in accessing housing, but little is known about its effects on diabetes outcomes, particularly in low- and middle-income countries.

**WHAT THIS STUDY ADDS**

- Our study is among the first to investigate the association between subsidised housing residency and a diabetes outcome among a low-middle-income country population. We found that subsidised housing residency was associated with a higher risk of diabetes mortality, and that the magnitude of the association was particularly higher in low socioeconomic status municipalities.

**HOW THIS STUDY MIGHT AFFECT RESEARCH, PRACTICE OR POLICY**

- Intensifying diabetes prevention and control actions and prompt treatment of the diabetes complications is warranted among subsidised housing residents, particularly among those living in low socioeconomic status municipalities.

## Introduction

Diabetes is a major public health problem that is more common among low socioeconomic groups.^1 2^ A growing body of research has called attention to the influence of housing access, one of the most significant social challenges for low socioeconomic groups, on diabetes outcomes such as incidence, hospitalisation, and glycaemic control.^1–4^ The association between housing access and diabetes outcomes can be explained by different mediators, with behavioural factors likely to be particularly important.^5^

Persons with diabetes can control their blood glucose levels by taking medication, following a diet, exercise, self-monitoring of blood glucose, and healthcare visits.^2 5^ These behaviours can be influenced by wider factors, including those related to housing.^1 2^ A qualitative study by Keene *et al*,^5^ conducted with 40 low-income adults with diabetes in New Haven, Connecticut, identified three mechanisms through which housing access may influence diabetes control behaviours. First, housing access may influence the prioritisation of diabetes management: challenges associated with homelessness and housing instability can consume emotional resources and physical energy, thus interfering with the ability to prioritise diabetes care.^3–5^ Second, housing access may influence the ability to establish and maintain diabetes management routines: sense of consistency and control associated with stable housing can support the adoption and maintenance of routines of diabetes control behaviours.^2–5^ Third, housing access may influence the ability to manage diabetes-related expenses: housing costs can compete with diabetes-related expenses, thus hindering diabetes management.^2 4 5^ Furthermore, even when medications and healthcare visits are fully covered by insurance or publicly funded health systems, there are still potential additional expenses, such as food for a diabetic diet.^2 5^ Moreover, not all diabetes medications may be covered by insurance or publicly funded health systems.^6^

Access to housing, in particular purchasing a home to achieve housing stability, can be very difficult for low socioeconomic groups. The wages and job security of these groups are often not sufficient to afford a home or to qualify for mortgage loans.^5^ However, there are countries that offer subsidies to low socioeconomic groups for home purchase. This is the case of Brazil, which has a particularly high level of housing shortage, in addition to a high diabetes burden. In 2017, the housing shortage in Brazil reached 7.8 million housing units, the highest in the country’s history.^7^ In 2019, the country was ranked first in Latin America and fifth in the world in number of adults with diabetes, with 16.8 million cases.^8^ Given that housing can support diabetes control and prevention behaviours,^5^ it is possible that subsidised housing in Brazil has been contributing to alleviate not only the housing shortage, but also the diabetes burden. To elucidate this, empirical assessment is necessary, especially considering that diabetes is a condition of complex multifactorial aetiology.^1^ Furthermore, a significant body of population-based studies have reported risk associations between subsidised housing residency and health,^9–13^ which reinforces the need for empirical assessment.

Diabetes mortality is related to diabetes incidence and management,^2 14^ with the Brazilian mortality registry providing a consolidated source of health data with national coverage^15^ and is therefore particularly suitable for study. Using Brazilian administrative records of social programs and death certificates, we aimed to analyse the association between subsidised housing residency and diabetes mortality among a cohort of low-income adults. Subsidised housing residency, the study exposure, assesses residency in housing subsidised by the Brazilian Federal Government through the Minha Casa Minha Vida (MCMV) programme,^16^ one of the largest in Latin America.

## Methods

### Study design

This is a retrospective cohort study of adults aged 18–79 years in Brazil who registered in Cadastro Único (CadÚnico) from 1 January 2010 to 31 December 2015. CadÚnico is a national administrative database of individuals applying for government social programmes.^17^ For registering in CadÚnico, individuals should belong to a family with a monthly income up to half a minimum wage per member, or up to three minimum wages in total.^17^ The minimum wage ranged from BR$510 (US$94.44) in 2010 to BR$788 (US$145.93) in 2015 (conversion of BR$ to US$ used the exchange rate of US$1=BR$5.40).^18^ Study participants entered the cohort at the date of registration in CadÚnico and were followed until occurrence of a diabetes death, the outcome, or until censoring due to a non-diabetes death or being alive by the end of the study period. The cohort was analysed longitudinally using survival analysis, which allowed accounting for the participants’ variable periods of follow-up and for the time-varying exposure (subsidised housing residency, the exposure, only starts at some time point after registration in CadÚnico). We minimised potential bias from non-random allocation of the exposure and differences between exposure groups through covariate balancing with inverse probability of treatment weighting combined with regression adjustment.^19 20^ Careful data cleaning and checking were performed to ensure data accuracy.

### Data sources and selection of the cohort participants

**Figure 1:**
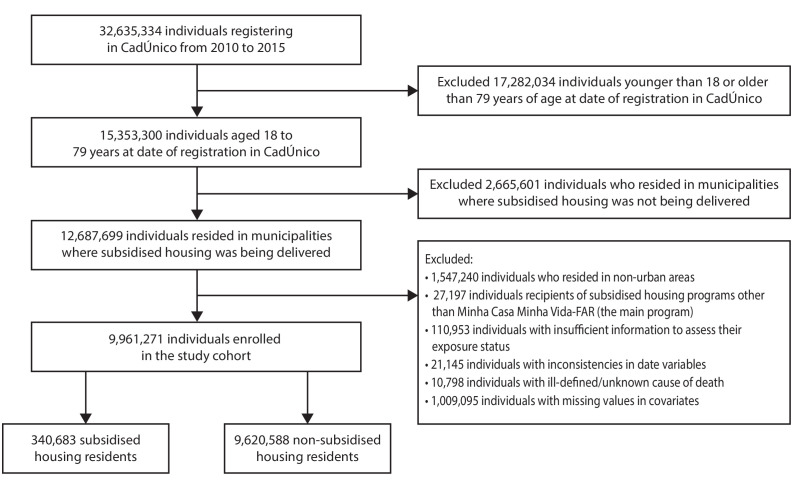
Study population flow chart. CadÚnico, Cadastro Único; FAR, Fundo de Arrendamento Residencial.

The individuals that comprised the study cohort were selected from records of the CadÚnico database, which was provided by the Brazilian Ministry of Citizenship. Two additional databases were linked^21 22^ to the CadÚnico database: the Sistema de Informação sobre Mortalidade, which was provided by the Brazilian Ministry of Health and included national records of death certificates, and a database with national records of subsidised housing recipients, provided by the Brazilian Ministry of Cities. The linked database contained records of 32 635 334 individuals who registered in CadÚnico during the study period (figure 1). Of those, 12 687 699 individuals were aged 18–79 years and resided in municipalities where subsidised housing was being delivered. We excluded individuals with missing data in covariates, individuals with inconsistencies in date variables, individuals with ill-defined/unknown cause of death, individuals who resided in non-urban areas, individual recipients of subsidised housing programmes other than MCMV-Fundo de Arrendamento Residencial (FAR) (the main programme, described below), and individuals with insufficient information to assess their subsidised housing residency status (i.e., individuals recorded in the subsidised housing database, but with no information on the date of subsidised housing receipt or the name of the subsidised housing programme). After these exclusions, 9 961 271 individuals remained in the study database for analysis.

### Diabetes mortality

Diabetes mortality, the study outcome, included diabetes both as the underlying or a contributory cause of death. Diabetes as a contributory cause was included as this is the most common type of reporting of diabetes on death certificates (persons with diabetes die more commonly from the complications of diabetes (e.g., stroke), not the disease itself).^14 23^ Diabetes as the underlying or a contributory cause of death was defined based on the diabetes International Classification of Diseases 10th Revision codes reported on the death certificates of the cohort participants: E10–E14 and O24 (online supplemental tables S1 and S2). We did not perform analyses for specific types of diabetes, such as type 1 and type 2 diabetes, due to limited reporting of the specific types of diabetes. For performing survival analysis, diabetes mortality was measured as time to death, defined as the number of days between the date of registration in CadÚnico and the date of a diabetes death.

10.1136/bmjdrc-2022-003224.supp1 Supplementary data

### Subsidised housing residency

The study exposure was subsidised housing residency. Subsidised housing residents are defined as individuals who reside in housing subsidised by the Brazilian Federal Government through the MCMV programme.^16^ Subsidised housing residents receive subsidised housing at some time point after registering in CadÚnico. The follow-up period of subsidised housing residents can therefore be divided into two periods: (1) from registering in CadÚnico to receiving subsidised housing (i.e., the time period when the exposure has not yet been assigned) and (2) from receiving subsidised housing onwards (i.e., the time period when the exposure has been assigned). To account for these two time periods in our survival analysis, subsidised housing residency was modeled as a time-dependent variable. Participants who do not receive subsidised housing were considered the control group.

The MCMV programme was created in 2009 by the Brazilian Federal Government to support the low-income population in accessing housing.^16^ Generally, housing units are produced specifically for the MCMV programme and subsidies are passed on to an MCMV recipient in the form of lower prices and interest rates: the programme can cover up to 90% of a home’s price, with annual interest rates ranging from 0% to 8.16%.^16^ Financial aspects of the programme, such as the amount of subsidies conferred and the charging for the MCMV homes, are the responsibility of a government federal bank.^16^

The MCMV programme has different subprogrammes, with MCMV-FAR which is focused on urban housing being the most common,^24^ and hence used to define our exposure variable. The study period began in 2010, as this was the year in which the MCMV-FAR housing units began to be delivered. MCMV housing units are assigned randomly among MCMV applicants,^24^ but we could not analyse both the successful and unsuccessful applicants (i.e., a randomised sample), as only the successful applicants’ records were available in the database of subsidised housing provided for our study.

### Covariates

Covariates were baseline characteristics including age, sex, education level, self-reported race, receipt of social cash transfers, year of registration in CadÚnico (i.e., cohort entry year), macroregion of residence, municipality of residence population size, and municipality of residence Human Development Index (HDI).^25^ HDI is a composite measure of a population’s life expectancy, per-capita income, and average education level.^25^ Its scale ranges from 0 to 1 and can be categorised in five levels: very low (0.000–0.499), low (0.500–0.599), medium (0.600–0.699), high (0.700–0.799), and very high (0.800–1.000)^25^ (information on the calculation of the municipality HDI is presented in online supplemental file 1).

### Statistical analysis

First, we described the cohort participants’ baseline characteristics, subsidised housing residency status, death certificates, and age-standardised mortality rate of diabetes. The age-standardised mortality rate of diabetes was calculated with the direct method and using the WHO standard population age distribution.^26^

Second, we calculated inverse probability of treatment weights (IPTW)^19^ to balance the covariates between subsidised housing residents and non-residents. Covariate balance was assessed using standardised differences (see online supplemental file 1 for information on their calculation), with smaller values indicating better balance (a common cut-off point for considering a covariate with adequate balance is ≤0.10).^27 28^

Third, we used a Cox model with IPTW weighting and regression adjustment for the covariates to analyse the association between subsidised housing residency and time to diabetes mortality. We reported the HR and its 95% CI, and adjusted survival curves^29^ for subsidised housing residents and non-residents. This analysis was also performed by groups of municipality HDI to assess inequalities. The combined use of IPTW weighting and regression adjustment is one of the most robust methods for minimising bias from non-random exposure assignment in observational studies.^19 20^

Fourth, to assess the consistency of our estimates, we analysed the association between subsidised housing residency and time to diabetes mortality using two alternative survival models: a parametric survival model^30^ and the Fine-Gray model.^31^ Unlike the Cox model, which makes no assumptions about the distributional form of the baseline hazard function, parametric survival models assume the baseline hazard function follows a particular distribution.^30^ This particular distribution can be determined by comparing the fit of parametric models with different distributions to a set of data. We performed this comparison and found the Gompertz distribution providing the best fit to our data (online supplemental table S3). When parametric models properly fit the data, a more precise estimation of parameters can be achieved.^30 32^ The Fine-Gray model is a modified version of the Cox model that accounts for competing risks.^31^ This model was applied with deaths by non-diabetes causes defined as competing risks. The parametric and Fine-Gray models were adjusted for the study covariates. A mathematical description of the three survival models used and the respective analysis codes are presented in online supplemental file 1.

Analyses were performed using the statistical software R V.3.6 and STATA V.15.1.

### Patient and public involvement

Patients and/or the public were not involved in the design, conduct or reporting of this research.

## Results

At baseline, the mean age of the cohort was 40.3 years (SD 15.6 years), with a majority of women (58.4%) (table 1). During 29 238 920 person-years of follow-up, the number of deaths with diabetes as the underlying or a contributory cause was 18 775 (10.7% of total deaths) (table 2). When diabetes was reported on the death certificate as a contributory cause of death (11 279), the conditions most frequently reported as the underlying cause of death were cardiovascular disease (5127; 45.5%), diabetes (1914; 17.0%—which was reported both as the underlying and as a contributory cause of death), respiratory disease (1524; 13.5%), and neoplasm (976; 8.7%). The age-standardised mortality rate of diabetes was 74.0 per 100 000 person-years.

**Table 1:** Cohort baseline characteristics

Baseline characteristic	Non-subsidised housing residents (n=9 620 588)	Subsidised housing residents (n=340 683)	Overall (n=9 961 271)
Age (mean (SD) in years)	40.4 (15.6)	37.7 (14.0)	40.3 (15.6)
Sex (%)			
Men	4 010 615 (41.7)	130 675 (38.4)	4 141 290 (41.6)
Women	5 609 973 (58.3)	210 008 (61.6)	5 819 981 (58.4)
Education level (%)			
Primary or less	5 544 107 (57.6)	181 463 (53.3)	5 725 570 (57.5)
Secondary or more	4 076 481 (42.4)	159 220 (46.7)	4 235 701 (42.5)
Race (%)			
White	3 519 819 (36.6)	117 575 (34.5)	3 637 394 (36.5)
Black, mixed or indigenous	6 100 769 (63.4)	223 108 (65.5)	6 323 877 (63.5)
Receipt of social cash transfers (%)			
No	5 014 581 (52.1)	197 308 (57.9)	5 211 889 (52.3)
Yes	4 606 007 (47.9)	143 375 (42.1)	4 749 382 (47.7)
Cohort entry year (%)			
2010	1 267 608 (13.2)	56 358 (16.5)	1 323 966 (13.3)
2011	1 400 342 (14.6)	100 168 (29.4)	1 500 510 (15.1)
2012	2 383 468 (24.8)	84 613 (24.8)	2 468 081 (24.8)
2013	1 294 060 (13.5)	43 766 (12.8)	1 337 826 (13.4)
2014	1 913 767 (19.9)	42 781 (12.6)	1 956 548 (19.6)
2015	1 361 343 (14.2)	12 997 (3.8)	1 374 340 (13.8)
Macroregion of residence (%)			
South	1 112 370 (11.6)	38 686 (11.4)	1 151 056 (11.6)
Southeast	4 372 032 (45.4)	130 141 (38.2)	4 502 173 (45.2)
Central-west	989 140 (10.3)	40 204 (11.8)	1 029 344 (10.3)
Northeast	2 259 759 (23.5)	94 176 (27.6)	2 353 935 (23.6)
North	887 287 (9.2)	37 476 (11.0)	924 763 (9.3)
Municipality population (inhabitants) (%)			
<500 000	6 063 337 (63.0)	245 819 (72.2)	6 309 156 (63.3)
≥500 000	3 557 251 (37.0)	94 864 (27.8)	3 652 115 (36.7)
Municipality Human Development Index (%)			
Low or very low	546 130 (5.7)	11 662 (3.4)	557 792 (5.6)
Medium	1 739 166 (18.1)	73 043 (21.4)	1 812 209 (18.2)
High or very high	7 335 292 (76.2)	255 978 (75.1)	7 591 270 (76.2)

**Table 2:** Analysis of the association between subsidised housing residency and time to diabetes mortality among the study cohort observed from 2010 to 2015

	Non-subsidised housing residents	Subsidised housing residents	Overall
n	9 620 588	340 683	9 961 271
Follow-up person-years	28 516 461	722 459	29 238 920
Diabetes mortality events (n)	18 363	412	18 775
Age-standardised mortality rate of diabetes (per 100 000 person-years)	73.68	90.98	74.00
HR (95% CI)	1	1.17 (1.05–1.31)	–

HR obtained from a Cox model with inverse probability of treatment weighting and regression adjustment for age, sex, education level, race, receipt of social cash transfers, cohort entry year, macroregion of residence, municipality population size, and municipality Human Development Index.

Cohort participants contributed a total of 29 238 920 person-years of follow-up time with a mean follow-up duration of 2.9 years (SD 1.6 years). By the end of the study period (31 December 2015), 340 683 participants (3.4% of the cohort) had received subsidised housing. This represented an increase of 504.5% compared with the 56 358 participants who received subsidised housing in 2010, the first year of the study period. The total person-years of subsidised housing residents (time since subsidised housing receipt) was 722 459 (2.5% of total person-years). The mean time since subsidised housing receipt was 2.1 years (SD 1.4 years).

**Figure 2:**
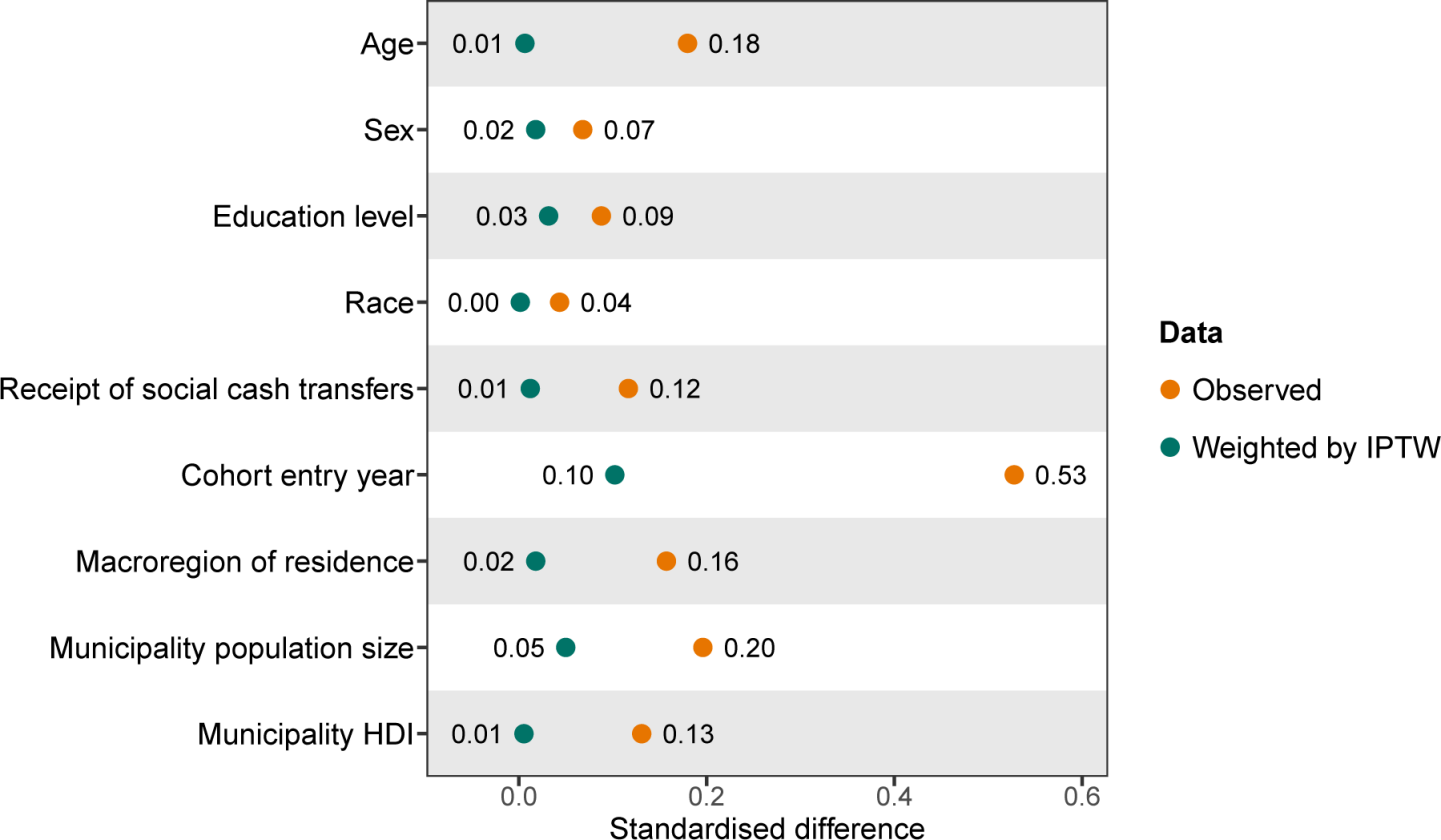
Standardised differences of baseline characteristics between subsidised housing residents and non-residents in the observed and IPTW-weighted data. HDI, Human Development Index; IPTW, inverse probability of treatment weights.

Standardised differences were smaller in the IPTW-weighted data than in the observed data (figure 2 and online supplemental table S4). Moreover, standardised differences in the IPTW-weighted data were less than or equal to 0.10, which is an indication of adequate covariate balance.

Subsidised housing residency was associated with a higher risk of diabetes mortality (HR 1.17; 95% CI 1.05 to 1.31) (table 2, online supplemental table S5 and figure S1). This association was also observed using a parametric model (HR 1.18; 95% CI 1.07 to 1.30, online supplemental table S6) and the Fine-Gray model (HR 1.19; 95% CI 1.08 to 1.32, online supplemental table S7).

The magnitude of the association between subsidised housing residency and time to diabetes mortality was more pronounced among participants of municipalities with medium, low or very low HDI (HR 1.30; 95% CI 1.04 to 1.62) than among participants of municipalities with high or very high HDI (HR 1.12; 95% CI 0.98 to 1.27) (table 3).

**Table 3:** Analysis of the association between subsidised housing residency and time to diabetes mortality among the study cohort observed from 2010 to 2015, by groups of municipality Human Development Index

	Participants of municipalities with medium, low or very low Human Development Index		Participants of municipalities with high or very high Human Development Index	
	Non-subsidised housing residents	Subsidised housing residents	Non-subsidised housing residents	Subsidised housing residents
n	2 285 296	84 705	7 335 292	255 978
Follow-up person-years	6 864 184	172 928	21 150 745	549 531
Diabetes mortality events (n)	4048	102	14 315	310
Age-standardised mortality rate of diabetes (per 100 000 person-years)	67.13	95.62	75.86	89.31
HR (95% CI)	1	1.30 (1.04–1.62)	1	1.12 (0.98–1.27)

HR obtained from a Cox model with inverse probability of treatment weighting and regression adjustment for age, sex, education level, race, receipt of social cash transfers, cohort entry year, macroregion of residence, and municipality population size.

## Discussion

We found that subsidised housing residency was associated with a higher risk of diabetes mortality. This finding is in line with a significant body of studies on the association between subsidised housing residency and health. Digenis-Bury *et al* 9 found associations between subsidised housing residency and risk of chronic diseases, including diabetes, among adults in Boston, Massachusetts, in 2001 and 2003. Parsons *et al* 10 found associations between subsidised housing residency and risk of chronic diseases, including diabetes, among a nationally representative sample of American adults older than 50 years in 2006. Seng *et al* 11 found an association between subsidised housing residency and risk of all-cause mortality (an outcome that includes diabetes deaths) among adults in Singapore in 2012. Simning *et al* 12 found an association between subsidised housing residency and risk of mental illness (which can be a risk factor for diabetes^33^) among a nationally representative sample of African-American adults in 2001–2003. Mehta *et al* 13 found an association between subsidised housing residency and risk of asthma (which can be a prevalent condition in persons with diabetes^34^) among adults in Boston, Massachusetts, in 2010–2015. In general, the authors of these studies speculate that their findings may be partly explained by subsidised housing residents having lower socioeconomic status, experiencing poorer housing/neighbourhood conditions, and by subsidised housing units being mainly located in low socioeconomic areas.^9–13^ These explanations highlight, in summary, that subsidised housing residents are a more socially vulnerable group.

Poor housing/neighbourhood conditions have been reported in subsidised housing estates in Brazil, which may be a contributing factor for the observed higher risk of diabetes mortality among subsidised housing residents. Poor housing/neighbourhood conditions can influence diabetes outcomes, for example, by functioning as barriers to diabetes control and prevention behaviours.^1 2 35^ Although we had no data on housing/neighbourhood conditions, there are studies that evaluated the housing/neighbourhood conditions of subsidised housing estates in Brazil. For example, Carvalho and Stephan^36^ evaluated the housing/neighbourhood conditions of three subsidised housing estates in Viçosa, southeast Brazil, in 2014, finding poor conditions related to infrastructure, basic services, and access to health, education, and leisure facilities. Furthermore, residents of those subsidised housing estates reported better general living conditions in their previous neighbourhoods.^36^ Similar findings were observed by de Moura,^37^ which evaluated the housing/neighbourhood conditions of 32 subsidised housing estates in the metropolitan region of Natal, northeast Brazil, in 2013. Logsdon *et al* 38 evaluated the quality of six subsidised housing estate projects in Cuiabá, central-west Brazil, in 2012–2014, concluding that the quality of all evaluated projects is precarious; the authors highlighted that none of the projects had adequate circulation and service areas, in all rooms of the housing units at least one item of the minimum necessary furniture was missing, the shape of the roof did not allow expansion of the residence, and the construction method and the materials used made housing adjustments or changes very difficult.^38^ Poor housing/neighbourhood conditions such as those reported by Carvalho and Stephan,^36^ de Moura,^37^ and Logsdon *et al* 38 may be partly explained by subsidised housing estates in Brazil being commonly built in areas of low socioeconomic status, where urban infrastructure is often less developed.^36 37^

High housing costs, which may influence health-related expenses,^5^ may also be a contributing factor for the observed higher risk of diabetes mortality among subsidised housing residents. High housing costs were reported in a qualitative study by Pereira,^39^ conducted in a subsidised housing estate in Poços de Caldas, southeast Brazil, in 2016–2018. The author obtained reports of high housing costs that included expenses such as the monthly payment for the purchase of subsidised housing, condominium fees, property taxes, and electricity and water service bills. Pereira^39^ also obtained a report from a municipal housing director that 70% of subsidised housing residents had housing debts. Pereira^39^ also observed vacant housing units and obtained reports that persons move out of subsidised housing due to reasons such as high housing costs, difficulty in adaptation, remote location, and low contact with family and friends. It is also worth mentioning the study by Rocha,^40^ which analysed the effect of subsidised housing on employment in Rio de Janeiro and São José do Rio Preto, southeast Brazil, in 2011 and 2013, finding a reduced probability of formal employment among subsidised housing recipients compared with non-recipients. This finding is noteworthy, as employment can determine an individual’s income; therefore, it can also determine the ability to pay health and housing expenses.

High availability of unhealthy foods, which may influence diabetes control and prevention,^1 2^ may also be a contributing factor for the observed higher risk of diabetes mortality among subsidised housing residents. Low-income neighbourhoods tend to have poor access to supermarkets and healthy foods but abundant access to fast-food outlets and energy-dense foods.^1^ This is a pattern that has been observed in the context of subsidised housing in Brazil. Vicentim and Kanashiro^41^ mapped the commercial establishments near a subsidised housing estate in Londrina, south Brazil, in 2012–2014, finding that the most frequent commercial establishments were bars (29.4%), supermarkets (21.6%), and fast-food outlets (13.3%). This high frequency of bars and fast-food outlets potentially indicates a high availability of unhealthy foods.

The magnitude of the association between subsidised housing residency and diabetes mortality was more pronounced among participants of municipalities with medium, low or very low HDI than among participants of municipalities with high or very high HDI. Since the HDI is an area-level indicator of socioeconomic status,^25^ this finding can be explained by the fact that persons in areas with lower socioeconomic status are generally exposed to more adverse health contexts (e.g., more deprived urban infrastructure) compared with persons in areas with higher socioeconomic status.^25 42^ Another noteworthy finding was that when diabetes was reported as a contributory cause of death, the underlying cause of death most frequently reported was cardiovascular disease. This highlights the well-established association between diabetes and cardiovascular disease.^1 14^ It is also noteworthy that the most frequent diabetes coding on death certificates was E14, unspecified diabetes mellitus (online supplemental tables S1 and S2). This potentially reflects a well-known clinical difficulty: the classification of diabetes.^43 44^ For example, the distinction between type 1 and type 2 diabetes in clinical practice is not always obvious based on initial history, physical examination, and laboratory values at ﬁrst presentation.^43^

There are key strengths to this study. The cohort provided sufficient statistical power to detect associations, in addition to providing relevant population representativeness: the cohort comprised approximately 10% of the Brazilian population aged 18–79 years,^45^ the age range at baseline. Another key strength was the combined use of IPTW weighting and regression adjustment, which is a robust method for minimising potential unobserved biases in observational studies.^19 20^ The use of alternative survival models is also a notable methodological feature. Parametric models, which can provide a more precise estimation of parameters when proper data fit is achieved,^30 32^ and the Fine-Gray model, which can take into account competing risks,^31^ both produced similar results, thus showing consistency in our estimates. Lastly, we highlight that the study outcome and exposure are measures related to two urgent and current issues not only in Brazil, but worldwide: diabetes and housing access. Worldwide, there has been an increasing trend in rates of obesity-associated chronic conditions including, notably, diabetes and cardiovascular disease.^46 47^ Similarly, rents and property prices have been soaring in many cities around the world, representing a significant barrier to access housing.^48–50^

This study also has limitations. First, the study follow-up period may be considered short. However, it should be noted that the study exposure is still recent. Second, we had no data on relevant confounders such as behavioural factors, comorbidities, including diagnosis of diabetes, and housing/neighbourhood conditions. However, IPTW weighting and regression adjustment were used to balance all observed covariates between subsidised housing residents and non-residents and to minimise the potential influence of unobserved confounding.^19 20^ Third, the quality of recording of administrative data in Brazil may vary over time and across the country,^15 51^ which may be a relevant source of confounding. However, to account for temporal differences in the quality of recording of administrative data, we adjusted the analyses for the year of registration in CadÚnico, and to account for geographical differences, or more generally, to account for macrosocioeconomic inequalities, we adjusted the analyses for the macroregion of residence, the municipality population size, and the municipality HDI.

Subsidised housing has been key in reducing housing shortages among the low-income population in Brazil, with over 1 million housing units being delivered from 2009 to 2020.^24^ However, despite this success, poor housing/neighbourhood conditions have been reported.^36–39^ Poor housing/neighbourhood conditions have also been reported in subsidised housing in other countries such as Chile,^48^ India,^49^ and the USA.^50^ Considering that having a home as well as housing/neighbourhood conditions can influence diabetes outcomes,^1 2 5 35^ it is therefore important that subsidised housing programmes not only deliver homes in quantity, but also in quality,^36–38^ including physical and social contexts that support health promotion and disease control.^1 2^ Furthermore, it is important that subsidised housing programmes also seek to support greater housing stability, which may also influence diabetes outcomes.^5 52^ Achieving housing stability is often difficult for subsidised housing residents due to their low socioeconomic status, which can limit their ability to pay housing expenses, even while receiving government subsidies.^39^

Since subsidised housing estates in Brazil are commonly located in socially disadvantaged areas,^36 37 39 41^ which are a potential predictor of poor diabetes management,^1 2^ contextual-level interventions may be warranted. Improvements in cardiometabolic health due to a contextual-level intervention were observed, for example, in the study by Gary-Webb *et al*,^53^ which conducted a natural experiment to analyse the effect of neighbourhood investment on cardiometabolic risk factors among a randomly selected cohort of residents from two low-income and predominantly African-American matched neighbourhoods, in 2016–2018. The authors found that residents from the neighbourhood that received more publicly funded investments (housing and commercial investment) showed improvements in hemoglobin A1c and high-density lipoprotein cholesterol levels compared with residents from the neighbourhood that received less investment.^53^

## Conclusion

This study showed that subsidised housing residents had a greater risk of diabetes mortality, particularly those living in low socioeconomic status municipalities. Further research is warranted to assess the individual and contextual factors that contribute to diabetes mortality among subsidised housing residents, as this may aid in formulating better policies to improve the health of this population. Since the subsidised housing programme used to define subsidised housing residency is still recent, further research is also warranted to assess the long-term effect of subsidised housing residency. Finally, the results presented in this study suggest the need to intensify diabetes prevention and control actions and prompt treatment of the diabetes complications among subsidised housing residents, particularly among those living in low socioeconomic status municipalities.

10.1136/bmjdrc-2022-003224.supp2 Supplementary data

## Data Availability

The data supporting this study were obtained from the Brazilian Ministry of Health, Ministry of Citizenship, and Ministry of Cities. Due to privacy regulations, restrictions apply to access these data. However, the authors are willing to make every effort to grant data availability upon reasonable request and express permission from the data provider institutions.
