# Supplementary material for: Subsidised housing and diabetes mortality: a retrospective cohort study of 10 million low-income adults in Brazil
Source: BMJ Open Diabetes Res Care. 2023 Jun 22;11(3):e003224. doi: 10.1136/bmjdrc-2022-003224 (PMC10314413; doi:10.1136/bmjdrc-2022-003224)
Supplement: Supplementary data [file bmjdrc-2022-003224supp001.pdf]

Supplemental Materials

Table S1. Frequency of reporting of diabetes as the underlying cause of death on the death certificates of the cohort participants.

Diabetes ICD-10 coding	Non-subsidised housing residents	Subsidised housing residents
E10: Type 1 diabetes mellitus	477	10
E11: Type 2 diabetes mellitus	844	13
E12: Malnutrition-related diabetes mellitus	49	0
E13: Other specified diabetes mellitus	36	0
E14: Unspecified diabetes mellitus	7,769	210
O24: Diabetes mellitus in pregnancy, childbirth, and the puerperium	2	0

Table S2. Frequency of reporting of diabetes as a contributory cause of death on the death certificates of the cohort participants.

Diabetes ICD-10 coding	Non-subsidised housing residents	Subsidised housing residents
E10: Type 1 diabetes mellitus	320	7
E11: Type 2 diabetes mellitus	1,103	22
E12: Malnutrition-related diabetes mellitus	5	0
E13: Other specified diabetes mellitus	12	0
E14: Unspecified diabetes mellitus	9,613	192
O24: Diabetes mellitus in pregnancy, childbirth, and the puerperium	5	0

Flores-Ortiz R, *et al. BMJ Open Diab Res Care* 2023; 11:e003224. doi: 10.1136/bmjdrc-2022-003224

Table S3. Goodness of fit statistics of survival parametric models with different distribution specifications.

Distribution	Log Likelihood	AIC	BIC
Exponential	-130,326.3	260,690.6	260,959.4
Weibull	-130,240.0	560,520.0	260,802.9
Gompertz	-130,217.6	260,475.2	260,758.2
Lognormal	-130,260.1	260,560.2	260,843.1
Loglogistic	-130,232.6	260,505.2	260,788.1

AIC, Akaike information criterion; BIC, Bayesian information criterion. Models were adjusted for age, sex, education level, race, receipt of social cash transfers, cohort entry year, macroregion of residence, municipality population size, and municipality Human Development Index.

Table S4. Cohort baseline characteristics by subsidised housing residency status.

Baseline characteristic	Observed data			Data weighted by IPTW		
	Non-subsidised housing residents	Subsidised housing residents	SD	Non-subsidised housing residents	Subsidised housing residents	SD
Age (mean (standard deviation) in years)	40.4 (15.6)	37.7 (14.0)	0.18	40.3 (15.6)	40.4 (14.9)	0.01
Sex			0.07			0.02
Men	4,010,615 (41.7%)	130,675 (38.4%)		3,999,722 (41.6%)	139,588 (42.5%)	
Women	5,609,973 (58.3%)	210,008 (61.6%)		5,620,708 (58.4%)	189,171 (57.5%)	
Education level			0.09			0.03
Primary or less	5,544,107 (57.6%)	181,463 (53.3%)		5,530,017 (57.5%)	194,112 (59.0%)	
Secondary or more	4,076,481 (42.4%)	159,220 (46.7%)		4,090,412 (42.5%)	134,646 (41.0%)	
Race			0.04			0.00
White	3,519,819 (36.6%)	117,575 (34.5%)		3,513,042 (36.5%)	119,810 (36.4%)	
Black, mixed, or indigenous	6,100,769 (63.4%)	223,108 (65.5%)		6,107,388 (63.5%)	208,948 (63.6%)	
Receipt of social cash transfers			0.12			0.01
No	5,014,581 (52.1%)	197,308 (57.9%)		5,033,442 (52.3%)	174,001 (52.9%)	
Yes	4,606,007 (47.9%)	143,375 (42.1%)		4,586,988 (47.7%)	154,758 (47.1%)	
Cohort entry year			0.53			0.10
2010	1,267,608 (13.2%)	56,358 (16.5%)		1,278,609 (13.3%)	44,006 (13.4%)	
2011	1,400,342 (14.6%)	100,168 (29.4%)		1,448,996 (15.1%)	51,001 (15.5%)	
2012	2,383,468 (24.8%)	84,613 (24.8%)		2,383,702 (24.8%)	85,496 (26.0%)	
2013	1,294,060 (13.5%)	43,766 (12.8%)		1,292,098 (13.4%)	46,277 (14.1%)	
2014	1,913,767 (19.9%)	42,781 (12.6%)		1,889,689 (19.6%)	67,486 (20.5%)	
2015	1,361,343 (14.2%)	12,997 (3.8%)		1,327,335 (13.8%)	34,493 (10.5%)	
Macroregion of residence			0.16			0.02
South	1,112,370 (11.6%)	38,686 (11.4%)		1,111,671 (11.6%)	36,890 (11.2%)	
Southeast	4,372,032 (45.4%)	130,141 (38.2%)		4,348,408 (45.2%)	149,661 (45.5%)	
Central-west	989,140 (10.3%)	40,204 (11.8%)		994,049 (10.3%)	33,325 (10.1%)	
Northeast	2,259,759 (23.5%)	94,176 (27.6%)		2,273,293 (23.6%)	79,215 (24.1%)	
North	887,287 (9.2%)	37,476 (11.0%)		893,009 (9.3%)	29,668 (9.0%)	
Municipality population (inhabitants)			0.20			0.05
< 500,000	6,063,337 (63.0%)	245,819 (72.2%)		6,093,406 (63.3%)	216,080 (65.7%)	
≥ 500,000	3,557,251 (37.0%)	94,864 (27.8%)		3,527,024 (36.7%)	112,678 (34.3%)	
Municipality Human Development Index			0.13			0.01
Low or very low	546,130 (5.7%)	11,662 (3.4%)		538,729 (5.6%)	18,136 (5.5%)	
Medium	1,739,166 (18.1%)	73,043 (21.4%)		1,750,122 (18.2%)	60,363 (18.4%)	
High or very high	7,335,292 (76.2%)	255,978 (75.1%)		7,331,579 (76.2%)	250,259 (76.1%)	

IPTW, inverse probability of treatment weights; SD, standardised difference.

Table S5. Analysis of the association between subsidised housing residency and time to diabetes mortality among the study cohort observed from 2010 to 2015, using a Cox model with inverse probability of treatment weighting.

	Hazard Ratio	95% Confidence Interval	P-value
Subsidised housing residency (yes vs no)	1.17	1.05–1.31	0.01
Covariate			
Age (per year)	1.10	1.10–1.10	< 0.01
Sex (women vs men)	0.83	0.81–0.85	< 0.01
Education level (secondary or more vs primary or less)	0.67	0.64–0.70	< 0.01
Race (black, mixed, or indigenous vs white)	1.13	1.09–1.16	< 0.01
Receipt of social cash transfers (yes vs no)	1.08	1.04–1.12	< 0.01
Cohort entry year (reference: 2010)			
2011	1.07	1.02–1.12	< 0.01
2012	1.11	1.06–1.16	< 0.01
2013	1.05	0.99–1.12	0.12
2014	0.98	0.92–1.05	0.57
2015	1.06	0.95–1.17	0.29
Macroregion of residence (reference: South)			
Southeast	0.87	0.84–0.91	< 0.01
Central-west	0.84	0.79–0.89	< 0.01
Northeast	0.88	0.83–0.93	< 0.01
North	0.73	0.68–0.78	< 0.01
Municipality population (≥ 500,000 vs < 500,000)	1.09	1.06–1.13	< 0.01
Municipality Human Development Index (reference: Low or very low)			
Medium	1.11	1.03–1.19	< 0.01
High or very high	1.23	1.14–1.33	< 0.01

Table S6. Analysis of the association between subsidised housing residency and time to diabetes mortality among the study cohort observed from 2010 to 2015, using a parametric survival model with the specification of the Gompertz distribution.

	Hazard Ratio	95% Confidence Interval	P-value
Subsidised housing residency (yes vs no)	1.18	1.07–1.30	0.01
Covariate			< 0.01
Age (per year)	1.10	1.10–1.10	< 0.01
Sex (women vs men)	0.83	0.81–0.85	< 0.01
Education level (secondary or more vs primary or less)	0.67	0.64–0.70	< 0.01
Race (black, mixed, or indigenous vs white)	1.13	1.09–1.16	< 0.01
Receipt of social cash transfers (yes vs no)	1.09	1.05–1.13	< 0.01
Cohort entry year (reference: 2010)			
2011	1.06	1.01–1.11	0.01
2012	1.11	1.06–1.16	< 0.01
2013	1.05	0.99–1.12	0.09
2014	0.99	0.93–1.06	0.87
2015	1.07	0.97–1.19	0.18
Macroregion of residence (reference: South)			
Southeast	0.87	0.83–0.91	< 0.01
Central-west	0.84	0.79–0.89	< 0.01
Northeast	0.88	0.83–0.93	< 0.01
North	0.72	0.67–0.78	< 0.01
Municipality population (≥ 500,000 vs < 500,000)	1.10	1.06–1.14	< 0.01
Municipality Human Development Index (reference: Low or very low)			
Medium	1.11	1.03–1.19	< 0.01
High or very high	1.23	1.14–1.32	< 0.01

Flores-Ortiz R, *et al. BMJ Open Diab Res Care* 2023; 11:e003224. doi: 10.1136/bmjdrc-2022-003224

Table S7. Analysis of the association between subsidised housing residency and time to diabetes mortality among the study cohort observed from 2010 to 2015, using the Fine-Gray model.

	Hazard Ratio	95% Confidence Interval	P-value
Subsidised housing residency (yes vs no)	1.19	1.08–1.32	< 0.01
Covariate			< 0.01
Age (per year)	1.10	1.10–1.10	< 0.01
Sex (women vs men)	0.85	0.82–0.87	< 0.01
Education level (secondary or more vs primary or less)	0.67	0.63–0.70	< 0.01
Race (black, mixed, or indigenous vs white)	1.12	1.09–1.16	< 0.01
Receipt of social cash transfers (yes vs no)	1.08	1.04–1.13	< 0.01
Cohort entry year (reference: 2010)			
2011	1.03	0.98–1.08	0.23
2012	1.06	1.01–1.11	0.01
2013	0.99	0.93–1.05	0.66
2014	0.91	0.86–0.97	< 0.01
2015	0.97	0.88–1.08	0.61
Macroregion of residence (reference: South)			
Southeast	0.88	0.84–0.92	< 0.01
Central-west	0.84	0.79–0.90	< 0.01
Northeast	0.89	0.84–0.94	< 0.01
North	0.73	0.68–0.79	< 0.01
Municipality population (≥ 500,000 vs < 500,000)	1.10	1.06–1.14	< 0.01
Municipality Human Development Index (reference: Low or very low)			
Medium	1.10	1.02–1.19	0.01
High or very high	1.22	1.13–1.31	< 0.01

Flores-Ortiz R, *et al. BMJ Open Diab Res Care* 2023; 11:e003224. doi: 10.1136/bmjdrc-2022-003224

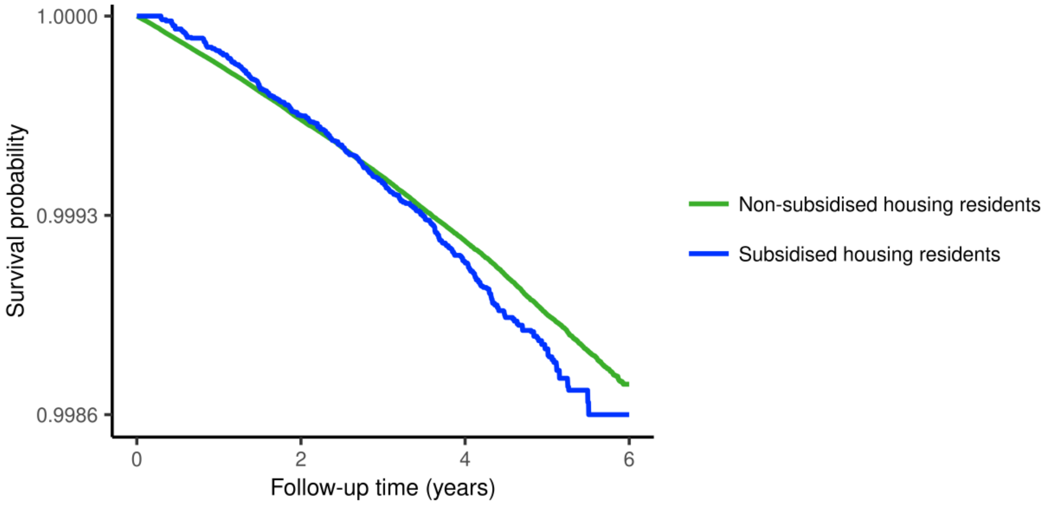

Figure S1. Adjusted survival functions of diabetes mortality for subsidised housing residents and non-residents. Survival functions obtained from a Cox model with inverse probability of treatment weighting and regression adjustment for age, sex, education level, race, receipt of social cash transfers, cohort entry year, macroregion of residence, municipality population size, and municipality Human Development Index.

Calculation of the Municipality Human Development Index

The Municipality Human Development Index is calculated by the geometric mean of three municipality-level variables: longevity, income, and education; using data from the 2010 Brazilian Census[1].

The longevity variable measures life expectancy, which is the average number of years that a person born in a certain municipality would live from birth[1]. It is calculated using demographic indirect methods[1].

The income variable measures the average income of residents of a given municipality[1]. It is calculated by the sum of income of all residents, divided by the number of people who live in the municipality[1].

The education variable is calculated by a weighted geometric mean of two variables[1]. The first variable, which has the weight of one, is the percentage of individuals aged 18 years or older that completed elementary school education[1]. The second variable, which has the weight of two, is the arithmetic mean of: (i) the percentage of individuals aged 5 to 6 years attending school, (ii) the percentage of individuals aged 11 to 13 years attending the final years of elementary school, (iii) the percentage of individuals aged 15 to 17 years with complete elementary school education, and (iv) the percentage of individuals aged 18 to 20 years with complete high school education[1].

Calculation of the standardised difference

The standardised difference was calculated in R using the "tableone" package[2]. This package uses the definitions of standardised difference described in Flury and Riedwyl[3] for continuous variables, Austin[4] for binary variables, and Yang et al.[5] for multinomial variables. Bellow are these definitions as described in Yang et al.[5].

- **Continuous variable**

For a continuous variable, the standardised difference is

$$d = \frac{(\overline{x_1} - \overline{x_2})}{\sqrt{\frac{s_1^2 + s_2^2}{2}}}$$

where $\overline{x_1}$ and $\overline{x_2}$ denote the sample mean of a baseline variable in each group, and s_1^2 and s_2^2 denote the sample variances, respectively.

- **Binary categorical variable**

For a binary categorical variable, the standardised difference is

$$d = \frac{(\hat{p}_1 - \hat{p}_2)}{\sqrt{\frac{\hat{p}_1(1 - \hat{p}_1) + \hat{p}_2(1 - \hat{p}_2)}{2}}}$$

where $\hat{p}_1$ and $\hat{p}_2$ denote the proportion or mean of a binary baseline variable in the treatment and control group, respectively.

- **Multinomial categorical variable**

For categorical baseline variables with K levels, Dalton[6] proposed to use a multivariate Mahalanobis distance method to generalize the standardised difference metric to handle a multinomial sample:

Let

$$T = \left(\hat{P}_{12}, \hat{P}_{13}, \dots, \hat{P}_{1K}\right)'$$

$$C = \left(\hat{P}_{22}, \hat{P}_{23}, \dots, \hat{P}_{2K}\right)'$$

where $\hat{P}_{jk} = Pr(category\ k|treatment\ group\ j),\ j \epsilon \{1, 2\}, and\ k \epsilon \{2, 3, \dots, K\}$.

The standardized difference is then defined as

$$d = \sqrt{(T - C)'S^{-1} + (T - C)}$$

where S is a $(k - 1) \times (k - 1)$ covariance matrix defined as:

$$S = [S_{kl}] = \begin{cases} \dfrac{\hat{P}_{1k}\left(1 - \hat{P}_{1k}\right) + \hat{P}_{2k}\left(1 - \hat{P}_{2k}\right)}{2}, & k = l \\ \dfrac{\hat{P}_{1k}\hat{P}_{1l} + \hat{P}_{2k}\hat{P}_{2l}}{2}, & k \neq l \end{cases}$$

Flores-Ortiz R, *et al. BMJ Open Diab Res Care* 2023; 11:e003224. doi: 10.1136/bmjdrc-2022-003224

Definition of the survival models used in the study

- **Cox model**

$$h(t,X) = h_0(t)e^{\sum_{i=1}^{p}\beta_i X_i}$$

h is the hazard at time *t* given a set of explanatory variables $X=(X_1, X_2, \ldots, X_p)$ [7];

$h_0(t)$ is the baseline hazard function [7].

- **Gompertz parametric model**

$$h(t,X) = e^{\gamma t}e^{\sum_{i=1}^{p}\beta_i X_i}$$

h is the hazard at time *t* given a set of explanatory variables $X=(X_1, X_2, \ldots, X_p)$ [8];

$e^{\gamma t}$ is the baseline hazard function [8];

γ is the shape parameter [8].

- **Fine-Gray model**

$$\lambda_r(t,X) = \lambda_{r0}(t)e^{\beta_r^T X}$$

λ is the subdistribution hazard of cause *r* for a subject with covariate vector X [9];

$\lambda_{r0}(t)$ is the baseline subdistribution hazard of cause *r*, and β_r is the vector of coefficients for the covariates [9].

Flores-Ortiz R, *et al. BMJ Open Diab Res Care* 2023; 11:e003224. doi: 10.1136/bmjdrc-2022-003224

Codes used to apply the study's survival models

- **Cox model (performed in R)**

```
library(survival)

library(survey)

iptw_data<-svydesign(ids=~1, weights=~iptw, data=observed_data)

cox_model<-svycoxph( Surv(time=tstart, time2=tstop, event=diabetesmort, type=”counting”)
~ subsidised_housing + age + sex + education + race + social_transfers + cohort_entry_year
+ municipality_population + municipality_hdi + municipality_region, design=iptw_data)

summary(cox_model)
```

- **Parametric model (performed in STATA)**

```
stset tstop, id(subject) failure(diabetesmort)

streg i.subsidisedhousing age i.sex i.educ i.race i.socialtranfers i.cohortentryyear
i.municipalitypopulation i.municipalityhdi i.municipalityregion, distribution(gompertz)
```


Flores-Ortiz R, *et al. BMJ Open Diab Res Care* 2023; 11:e003224. doi: 10.1136/bmjdrc-2022-003224

- **Fine-Gray model (performed in STATA)**

```
stset tstop, id(subject) failure(diabetesmort)

stcrr i.subsidisedhousing age i.sex i.educ i.race i.socialtranfers i.cohortentryyear i.municipalitypopulation i.municipalityhdi i.municipalityregion, compete(othercauses) noshow nolog
```

REFERENCES

1 PNUD, IPEA e FJP. O Índice de Desenvolvimento Humano Municipal Brasileiro. Brasília: 2013.

2 Yoshida K. tableone: Create ‘Table 1’ to Describe Baseline Characteristics with or without Propensity Score Weights. https://cran.r-project.org/web/packages/tableone/

3 Flury BK, Riedwyl H. Standard Distance in Univariate and Multivariate Analysis. *Am Stat* 1986;**40**:249. doi:10.2307/2684560

4 Austin PC. Using the Standardized Difference to Compare the Prevalence of a Binary Variable Between Two Groups in Observational Research. *Commun Stat - Simul Comput* 2009;**38**:1228–34. doi:10.1080/03610910902859574

5 Yang D, Dalton JE. A unified approach to measuring the effect size between two groups using SAS. In: *SAS Global Forum 2012*. 2012.

6 Dalton J. A new standardized difference metric for multinomial samples. *Unpubl Work* 2008.

7 Kleinbaum DG, Klein M. The Cox Proportional Hazards Model and Its Characteristics. 2012. 97–159. doi:10.1007/978-1-4419-6646-9_3

8 Kleinbaum DG, Klein M. Parametric Survival Models. 2012. 289–361. doi:10.1007/978-1-4419-6646-9_7

9 Scrucca L, Santucci A, Aversa F. Regression modeling of competing risk using R: an in depth guide for clinicians. *Bone Marrow Transplant* 2010;**45**:1388–95. doi:10.1038/bmt.2009.359
