# Supplementary material for: Subsidised housing and diabetes mortality: a retrospective cohort study of 10 million low-income adults in Brazil
Source: BMJ Open Diabetes Res Care. 2023 Jun 22;11(3):e003224. doi: 10.1136/bmjdrc-2022-003224 (PMC10314413; doi:10.1136/bmjdrc-2022-003224)
Supplement: Supplementary data [file bmjdrc-2022-003224supp002.pdf]

Reflexivity Statement

Domain	Question	Response
Study conceptualization	1. How does this study address local research and policy priorities?	The relationship between diabetes and socioeconomic factors, such as housing factors, is well established and remains a clear research priority to support policy planning in low-middle income countries. This has been evidenced by the interest that local partners have had in supporting this work.
	2. How were local researchers involved in study design?	The study design was developed collaboratively between Brazilian and UK researchers at face-to-face and virtual meetings.
Research management	3. How has funding been used to support the local research team?	Funding supported data safeguarding, processing, and analysis performed by Brazilian researchers.
Data acquisition and analysis	4. How are research staff who conducted data collection acknowledged?	We acknowledged in the manuscript the Brazilian Ministry of Health, Ministry of Citizenship, and Ministry of Cities for providing the data, and the data production team of CIDACS-FIOCRUZ for supporting with data linkage.
	5. How have members of the research partnership been provided with access to study data?	Study data has been made available to all researchers upon request and obtaining the security credentials from CIDACS-FIOCRUZ.
	6. How were data used to develop analytical skills within the partnership?	The data have been used for several studies at CIDACS-FIOCRUZ, contributing to the development of analytical skills of early career researchers.
Data interpretation	7. How have research partners collaborated in interpreting study data?	All authors met regularly to discuss the findings of this and other works.
Drafting and revising for intellectual content	8. How were research partners supported to develop writing skills?	RFO, the first author, wrote the first draft and all authors then contributed to the drafting and revision of the manuscript.
	9. How will research products be shared to address local needs?	Meetings and institutional communication actions are planned to disseminate the research locally.
Authorship	10. How is the leadership, contribution and ownership of this work by LMIC researchers recognised within the authorship?	Most of the authors are Brazilian, including the first author. The contributions of Brazilian researchers were recognised in the study.
	11. How have early career researchers across the partnership been included within the authorship team?	RFO, AJFF, CT, JP, and JFO are early career researchers.
	12. How has gender balance been addressed within the authorship?	9 authors are men and 10 are women.

Training	13. How has the project contributed to training of LMIC researchers?	The project included early career researchers who were trained to develop different research skills, including analysis and writing skills.
Infrastructure	14. How has the project contributed to improvements in local infrastructure?	The support from this project contributed to training of early career researchers and to improving CIDACS-FIOCRUZ data structures.
	15. What safeguarding procedures were used to protect local study participants and researchers?	The data analysed is stored at CIDACS-FIOCRUZ under strict security standards. Analyses were performed on de-identified data in a trusted research environment with access by VPN.
